# Alterations of Myelin Content in Parkinson’s Disease: A Cross-Sectional Neuroimaging Study

**DOI:** 10.1371/journal.pone.0163774

**Published:** 2016-10-05

**Authors:** Douglas C. Dean, Jitka Sojkova, Samuel Hurley, Steven Kecskemeti, Ozioma Okonkwo, Barbara B. Bendlin, Frances Theisen, Sterling C. Johnson, Andrew L. Alexander, Catherine L. Gallagher

**Affiliations:** 1 Waisman Center, University of Wisconsin Madison, Madison, Wisconsin, United States of America; 2 William S. Middleton Memorial Veterans Hospital, Madison, Wisconsin, United States of America; 3 Department of Neurology, University of Wisconsin Madison, Madison, Wisconsin, United States of America; 4 Oxford Centre for Functional Magnetic Resonance Imaging of the Brain, University of Oxford, Oxford, Oxfordshire, United Kingdom; 5 Wisconsin Alzheimer’s Disease Research Center, University of Wisconsin School of Medicine and Public Health, Madison, Wisconsin, United States of America; 6 Department of Medical Physics, University of Wisconsin School of Medicine and Public Health, Madison, Wisconsin, United States of America; 7 Department of Psychiatry, University of Wisconsin-Madison, Madison, Wisconsin, United States of America; Taipei Veterans General Hospital, TAIWAN

## Abstract

Alterations to myelin may be a core pathological feature of neurodegenerative diseases. Although white matter microstructural differences have been described in Parkinson's disease (PD), it is unknown whether such differences include alterations of the brain’s myelin content. Thus, the objective of the current study is to measure and compare brain myelin content between PD patients and age-matched controls. In this cross-sectional study, 63 participants from the Longitudinal MRI in Parkinson's Disease study underwent brain MRI, Unified Parkinson's Disease Rating Scale (UPDRS) scoring, and cognitive asessments. Subjects were imaged with the mcDEPSOT (multi-component driven equilibrium single pulse observation of T1 and T2), a multicomponent relaxometry technique that quantifies longitudinal and transverse relaxation rates (R_1_ and R_2_, respectively) and the myelin water fraction (VF_M_), a surrogate for myelin content. A voxel-wise approach was used to compare R_1_, R_2_, and VF_M_ measures between PD and control groups, and to evaluate relationships with age as well as disease duration, UPDRS scores, and daily levodopa equivalent dose. PD subjects had higher VF_M_ than controls in frontal and temporal white matter and bilateral thalamus. Greater age was strongly associated with lower VF_M_ in both groups, while an age-by-group interaction suggested a slower rate of VF_M_ decline in the left putamen with aging in PD. Within the PD group, measures of disease severity, including UPDRS, daily levodopa equivalent dose, and disease duration, were observed to be related with myelin content in diffuse brain regions. The age-by-group interaction suggests that either PD or dopaminergic therapies allay observed age-related myelin changes. The relationships between VF_M_ and disease severity measures suggests that VF_M_ may provide a surrogate marker for microstructural changes related to Parkinson’s disease.

## Introduction

Idiopathic Parkinson’s disease (PD) is an age-related neurodegenerative disease that is characterized by motor symptoms of tremor, rigidity, and bradykinesia, as well as non-motor symptoms affecting sleep, cognition, and autonomic function [1]. Motor symptoms correspond to cell loss in the substantia nigra pars compacta (SNc) and are improved by the use of dopamine agonist and precursor medications. Clinical symptoms are related to disruption of information flow through frontal-subcortical networks, which mediate motor, spatial, visual, and affective functions [2]. The anatomic substrate of these brain networks is, presumably, myelinated axons, which connect cell groups across cortical and subcortical brain regions [3–5], however, the contribution of myelin and its role of aging and neurodegeneration has only recently begun to be appreciated [6,7].

Athough PD is primarily considered a grey matter disease, recent investigations suggest that alterations in white matter may accompany or even play a role in the disease process. Cross-sectional pathological studies suggest that neurodegeneration in PD proceeds in a topographic sequence, first affecting the caudal brainstem and olfactory bulb, later in the SNc, followed by the thalamus and mesocortex, and finally neocortex [8]. It has been hypothesized that pathologic changes of PD progress in an inverse pattern to brain myelination, with thinly myelinated cortical projection neurons being selectively vulnerable [8]. Recent MRI techniques have enabled measurement of abnormalities in normal appearing white matter in a number of neurodegenerative and neuropsychiatric conditions, including PD. We and others have described alterations of white matter microstructural integrity across various brain regions, including in frontal white matter and brainstem, in PD using diffusion tensor imaging (DTI) [9–12]—however, while DTI is sensitive to alterations of myelin, it is additionally sensitive to other white matter microstructural changes [13,14]. Therefore, it is not known what microstructural elements are responsible for the observed diffusion signal differences in PD.

Moreover, an important factor that ought to be considered in studies of white matter is medications that modulate neurotransmission through frontal-subcortical circuits, which have been shown to affect cerebral white matter. Antipsychotic agents, which reduce dopaminergic neurotransmission, have been found to increase frontal white matter volume [15], while other medications used in the treatment of tremor in Parkinson’s disease, such as benztropine, promote remyelination [16]. These findings, as well as recent structural and functional MRI studies of individuals diagnosed with Parkinson’s disease, led us to postulate that alterations in brain myelin content may be detectable in PD.

In this cross-sectional neuroimaging study, we sought to examine MRI measures sensitive to white matter myelin content, comparing idiopathic Parkinson’s disease patients to typical, age-matched controls. To do so, we implemented a novel quantitative MRI technique known as multicomponent driven equilibrium single pulse observation of T_1_ and T_2_ (mcDESPOT) [17] to measure the longitudinal and transverse relaxation rates (R_1_ and R_2_, respectively), as well as the myelin water fraction (VF_M_), a surrogate for myelin content [17,18]. Our analyses tested whether (1) measures of myelin content differed between PD patients and age-matched controls, (2) group differences were influenced by age, and (3) neuroimaging measures of myelin content were related to clinical measures of disease duration and severity, including daily levodopa equivalent dose and Unified Parkinson’s Disease Rating Scale (UPDRS) scores [19].

## Materials and Methods

### Study Design and Participants

PD and control subjects were recruited through local movement disorders clinics and the Wisconsin Alzheimer’s Disease Research Center (ADRC) as part of a longitudinal MRI study of PD sponsored by the Department of Veterans Affairs, CS R&D. Study procedures included brain MR imaging, cognitive assessment, and UPDRS [19] scoring while off anti-Parkinson medication for 12–18 hours by a movement disorders neurologist (CG). Each participant provided written informed consent and the study was performed under guidelines approved by the University of Wisconsin-Madison’s institutional review board and the WS Middleton VA R&D committee.

Subjects were screened for cognitive impairment prior to enrollment using a competency questionnaire as well as the Mini Mental State Examination (MMSE [20]). Exclusion criteria included MMSE<27, other major central nervous system or medical diseases, and MRI ineligibility. PD subjects met UK brain bank criteria for idiopathic Parkinson’s disease [21] and were screened in detail for symptoms of atypical Parkinsonism such as supranuclear opthalmoparesis, falls, marked dysautonomia, axial rigidity, apraxia, and sensory neglect. Following MRI, 4 subjects were excluded from further analyses based on structural brain lesions. In total, data from 28 PD patients with a Hoehn and Yahr score between 1 and 3 and 35 age-matched controls were analyzed. Additional demographic and sample characteristics of the resulting cohort are described in Table 1. PD participants also provided history regarding the nature, location, and timing of motor symptom onset, which was used to calculate disease duration. All but 2 PD subjects were taking anti-Parkinson medications. For subjects taking anti-parkinson medications, daily levodopa equivalent doses were calculated from total daily dose according to a standard formula [22].

**Table 1:** Demographic characteristics of Parkinson’s disease (PD) and control subjects. Mean values are provided with standard deviations denoted in parentheses. PD patients were observed to differ from the control group in UPDRS total and and motor sub scores.

	PD Group	Control Group	P-value
Mean Age (years)	66.4 (9.9)	65.9 (7.4)	0.81
Sex (M/F)	23/5	24/11	0.21
Education (years)	16 (2.9)	17 (3)	0.11
UPDRS^a^ Motor Sub Score	20.0 (11.7)	1.7 (2.1)	**< .001**
UPDRS^a^ Total Score	34.9(17.7)	3.0(3.3)	**< .001**
Disease duration	6.4 (4.0)	N/A	N/A
Hoehn and Yahr Stage (Range)	1.6 (1–3)	0 (0)	N/A
Daily levodopa equivalent dose (mg)	443.4 (319)	0 (0)	N/A

^a^Unified Parkinson’s Disease Rating Scale, scored off anti-Parkinson medications for 12–18 hours.

N/A: Not applicable.

### MRI Data Acquisition

Imaging was performed between February, 2014 and April, 2015 on a 3.0 Tesla General Electric MR750 Discovery scanner (General Electric Healthcare, Waukesha, WI) equipped with an 8-channel head coil. Spoiled gradient recalled echo (SPGR, spoiled FLASH) and balanced steady-state free precession (bSSFP, TrueFISP) images were acquired over multiple flip angles, as part of the mcDESPOT protocol [17]. A common field of view of 25.6 cm × 25.6 cm × 16.8 cm and an isotropic voxel resolution of 2.0mm^3^ was shared amongst all images. Acquisition time was approximately 10 minutes per subject. The bSSFP data was acquired with two phase-cycling patterns (0° and 180°) to allow for correction of main (B_0_) magnetic field off-resonance [23] and a Bloch-Siegert B_1_ mapping technique [24] was used to correct for inhomogeneities of the transmit (B_1_) magnetic field.

### Image Processing

Imaging data were visually inspected for corrupting artifacts. Each subject’s SPGR, bSSFP, and Bloch-Siegert B_1_ map was first linearly coregistered to the high-flip angle (i.e. 18°) SPGR image in order to account for subtle head motion [25]. Non-parenchymal image voxels were removed [26]. VF_M_ maps were calculated by fitting the SPGR and bSSFP images to a three-component tissue model, which has been shown to provide improved characterization of brain microstructure and is less susceptible to partial volume effects [27]. R_1_ (1/T_1_) and R_2_ (1/T_2_) maps were additionally estimated from the imaging data [28].

Following calculation of these maps, participant’s R_1_, R_2_, and VF_M_ maps were nonlinearly aligned to the Montreal Neurological Institute (MNI) template. Using the Advanced Normalization Tools (ANTs) software package, a study-specific T_1_-weighted template was created using the high flip angle SPGR image from a representative sample of study participants [29–31]. Next, an affine transformation between the study-specific T_1_-weighted template and the MNI template was calculated [25].

**Fig 1:**
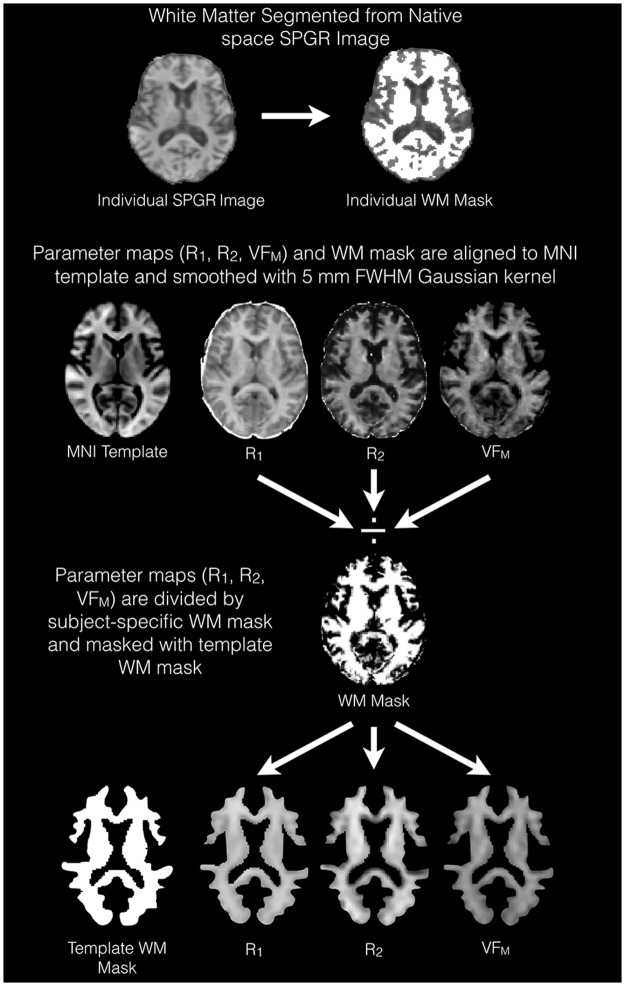
TSPOON Processing of relaxometry maps Illustrative diagram depicting the TSPOON processing used to help compensate for the smoothing of images and potential partial volume effects. A native-space white matter mask is first calculated from by segmenting subject’s high flip ange SPGR image. Parameter maps and the native-space white matter mask are then smoothed with a Gaussian kernel and the template-aligned parameter maps are divided by the white matter mask. Lastly, an overall white matter mask calculated from the population averaged VF_M_ map is applied to the relaxometry maps.

To improve the tissue specificity of the R_1_, R_2_, and VF_M_ measurements and minimize potential partial volume effects and mis-registration errors in subsequent analyses, parameter maps were masked and smoothed following the tissue-specific, smoothing-compensated (TSPOON) method [32]. This tissue and smoothing-compensated technique reduces the potential of morphological confounds between subjects and has been shown to be consistent with DTI analyses examining measures of anatomical regions of interest [32]. Briefly, a binary white matter mask was created for each participant by segmenting white matter from the high flip angle SPGR image using FMRIB’s Automated Segmentation Tool (FAST; [33]). This white matter mask and the quantitative parameter maps (i.e. R_1_, R_2_, and VF_M_) were then normalized to the population-specific template by applying the participant-specific spatial transformations estimated using ANTs in a single processing step to avoid multiple interpolations [34]. Spatially aligned masks and parameters were then smoothed with a 5mm full-width-at-half-max Gaussian kernel. Each participant’s smoothed R_1_, R_2_, and VF_M_ maps were next divided by the participant’s smoothed native-space white matter mask. Finally all parameter maps were masked by a population average white matter mask created by thresholding the average VF_M_ map at 0.05. Fig 1 illustrates this procedure. Subsequent analyses used these TSPOON-corrected VF_M_, R_1_, and R_2_ maps.

### Differences Between PD Patients and Age-Matched Controls

Linear regression models were developed to examine group differences of R_1_, R_2_, and VF_M_ between PD and age-matched controls. These regression models were constructed with R (Version 3.2.1) [35] and voxel-wise modeling was performed, controlling for age, sex, and years of formal education. Significance was defined using a two-stage procedure with first contingous clusters determined at p<0.005 (t>2.9). Next, the global smoothness of the regression residuals was estimated (3dFWHMx, AFNI, http://afni.nimh.nih.gov) and Monte Carlo simulations (3dClustSim, AFNI, http://afni.nimh.nih.gov) were used to estimate the minimum cluster-extent for significance when correcting for multiple comparisons using the family-wise error rate (FWE) [36]. Contiguous clusters of a minimum of 74 voxels were deteremined to be significiant (p<0.05). We additionally report areas found to be marginally significant (p<0.10), as these areas may be informative to underlying white matter microstructural changes in PD in a larger sample. Contiguous clusters of at least 50 voxels were considered to be marginally significant (p < 0.10) and all findings reported at least met this trend-level threshold p < 0.10.

Age-related changes in brain myelin across the lifespan have been described [34,37–41]. Thus, we additionally examined the effect of age on R_1_, R_2_ and VF_M_ and hypothesized that age-related associations may differ between PD patients and controls. Voxelwise linear regression models were constructed to examine the relationships with age and the age-by-group interaction, while accounting for the nuisance variables of sex and years of education. Here, the non-zero interaction term corresponds to age-related changes that differ between PD patients and controls. Significance was defined as p<0.05, cluster-corrected.

### Analysis of VF_M_, R_1_, and R_2_ Measurements

While the quantitative parameters are believed to be sensitized to underlying myelin, it is known that R_1_ and R_2_ are additionally sensitive to other biological alterations. In particular, R_1_ can be influenced by edema and inflammation [42], while R_2_ is susceptible to alterations of iron content [43], which is known to be influential in PD [44]. However, it is less clear whether biological influences that affect R_1_ or R_2_, could additionally influence alterations of VF_M_. To examine this, we performed a post-hoc analysis in which we re-analyzed the group differences and age-by-group interactions after incorporating voxelwise R_1_ and R_2_ measurements as additional nuisance covariates in the linear regression models. By including R_1_ and R_2_ measurements as additional regressors within these linear models, similar to a multimodal integrative image analysis framework [45], we are able to account for possible confounding effects R_1_ and R_2_ may have on the relationships with VF_M_.

### Associations Between Brain Imaging Parameters and Disease Severity Measures

Finally, we sought to investigate relationships between myelin content and measures of disease severity within the PD subject group. Specifically, associations of VF_M_ with disease duration, daily levodopa equivalent dose, and total UPDRS scores [19] were examined. First, a composite severity measure was created by averaging the standardized (i.e. z-score) disease severity measures and voxel-wise regression was used to examine the overall relationship between standardized VF_M_ and this composite disease severity score. Next, a separate regression model was constructed for each standardized disease severity measure to examine the associations between each of these measures and VF_M_. All models included standarized age, sex, years of formal education, and R_1_ and R_2_, as covariates, while significance was defined as p<0.05, cluster-corrected.

## Results

### Comparison of PD Patients and Age-Matched Controls

Quantitative R_1_, R_2_, and VF_M_ maps from 28 PD patients and 35 age-matched controls were used to assess group differences in white matter myelin content. The PD and control groups did not differ significantly by age, sex, or years of formal education, however, as expected, the PD group did have higher (p<0.001, uncorrected) UPDRS total and motor sub scores (Table 1).

#### Group Difference Analysis

**Fig 2:**
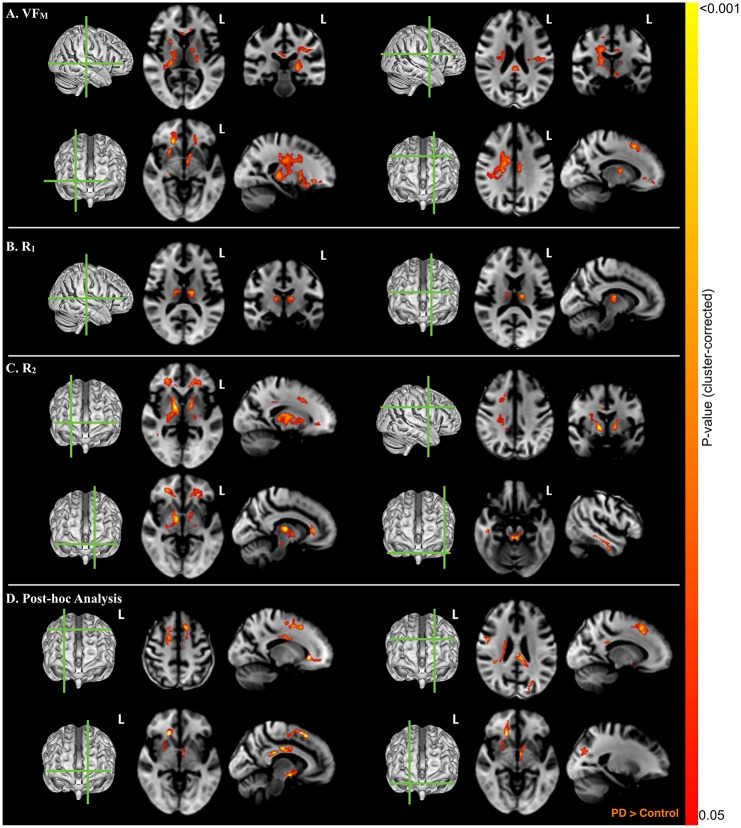
VF_M_, T_1_, T_2_ Differences between PD Subjects and Controls. (A) Compared with age-matched controls, PD subjects had higher VF_M_ in bilateral thalamus and posterior limb of the internal capsule, left inferior temporal gyrus, right superior longitudinal fasciculus, and portions of the genu of the corpus callosum (shown in red). (B) R_1_ was found to be be increased within the left and right thalmus of the PD group. (C) R_2_ was found to be increased in the PD group in superior and inferior frontal white matter, anterior limb of the internal capsule, and genu of corpus callosum, as well as portions of the anterior putamen and pallidum. (D) Re-analyzing the VF_M_ group differences with R_1_ and R_2_ as covariates yielded increased VF_M_ in superior frontal white matter and genu of the corpus callosum in the PD group. Statistical images are overlaid on the study-specific T1-weighted template and results are shown p<0.05, corrected for multiple comparisons at the cluster extent. An anatomical reference for each representative figure is additionally provided. The magnitude and atlas locations of maximally significant differences in regional VF_M_ are shown in Table 2.

Representative group differences between PD and age-matched control groups, (p<0.05, cluster-corrected) are shown in Fig 2, while cluster extents and locations are summarized in Table 2. The PD group displayed increased VF_M_ and R_2_ in the thalamic radiations and posterior limb of the internal capsule, right centrum semiovale encompassing superior longitudinal fasciculus, genu of corpus callosum and selected frontal and temporal regions. Compared to controls, PD patients also exihibited increased VF_M_ within the body of the corpus callosum and increased R_2_ in bilateral pallidum and anterior limb of the internal capsule. R_1_ in bilateral thalamus was also higher in PD patients than controls.

**Table 2:** Regions in which significant VF_M_ differences were observed between PD patients and age-matched controls. Abbreviations: R: Right; L: Left; WM: White Matter; Sup: Superior; Inf: Inferior; Ant: Anterior; Mid: Middle; Med: Medial; Lat: Lateral;

Image Modality	Location	Direction	MNI Coordinates			T-Statistic	P-Value	Size (mm^3^)
			X	Y	Z			
VF_M_	R. Centrum Semiovale	PD > Control	20	-18	28	3.64	0.0006	14552
	R. Ant. Corona Radiata and Genu of Corpus Callosum	PD > Control	18	30	-4	4.43	<0.0001	3560
	L. Post. Limb Internal Capsule	PD > Control	-16	-4	12	3.15	0.0026	1080
	L. Sup. Frontal Gyrus WM	PD > Control	-10	20	52	3.57	0.0007	992
	L. Ant. Thalamic Radiation	PD > Control	-6	-6	-12	3.44	0.0011	832
	L. Sup. Longitudinal Fasciculus	PD > Control	-48	-16	20	3.21	0.0022	784
	Splenium of Corpus Callosum	PD > Control	0	-32	20	3.36	0.0014	632
	Body of Corpus Callosum^a^	PD > Control	-8	-16	30	3.00	0.004	552
R_1_	L. Thalamus	PD > Control	-14	-14	12	3.43	0.0011	984
	R. Thalamus^a^	PD > Control	14	-14	10	3.21	0.0022	584
R_2_	R. Post. Limb Internal Capsule	PD > Control	10	-4	-2	4.03	0.0002	9464
	L. Thalamus	PD > Control	-10	-16	8	3.96	0.0002	3120
	Genu of Corpus Callosum	PD > Control	-6	24	6	3.63	0.0006	3096
	L. Inf. Fronto-Occcipital Fasciculus	PD > Control	26	44	-4	3.39	0.0013	1616
	Dorsal Midbrain	PD > Control	4	-32	-18	3.60	0.0007	1232
	R. Inf. Temporal Gyrus WM	PD > Control	50	-12	-34	3.71	0.0005	1088
	R. Sup. Frontal Gyrus WM	PD > Control	16	18	38	3.18	0.0024	704
	L. Sup. Parietal WM^a^	PD > Control	-20	-56	50	3.42	0.0011	408
Post-hoc	R. Sup. Corona Radiata	PD > Control	22	-18	28	3.67	0.0005	2408
	R. Sup. Frontal Gyrus WM	PD > Control	16	18	48	3.37	0.0013	2256
	L. Sup. Frontal Gyrus WM	PD > Control	-8	22	54	4.32	<0.0001	2024
	Splenium of Corpus Callosum	PD > Control	0	-34	18	4.01	0.0002	1792
	R. Ant. Corona Radiata and Genu of Corpus Callosum	PD > Control	18	30	-4	4.73	<0.0001	1736
	L. Ant. Thalamic Radiation	PD > Control	-8	-4	-12	3.81	0.00034	1000
	Cingulum	PD > Control	-8	-16	32	3.95	0.0002	888
	R. Sup. Longitudinal Fasiculus^a^	PD > Control	48	0	22	3.58	0.0007	584
	L. Lat. Occipital WM^a^	PD > Control	-20	-74	32	3.18	0.0023	560
	L. Mid. Temporal Gyrus WM^a^	PD > Control	-54	-26	-12	3.37	0.0013	552
	R. Sup. Longitudinal Fasiculus^a^	PD > Control	32	-20	36	3.18	0.0023	472

^a^Contiguous clusters of at least 50 voxels found to be marginally significant (p<0.1, cluster-corrected)

**Fig 3:**
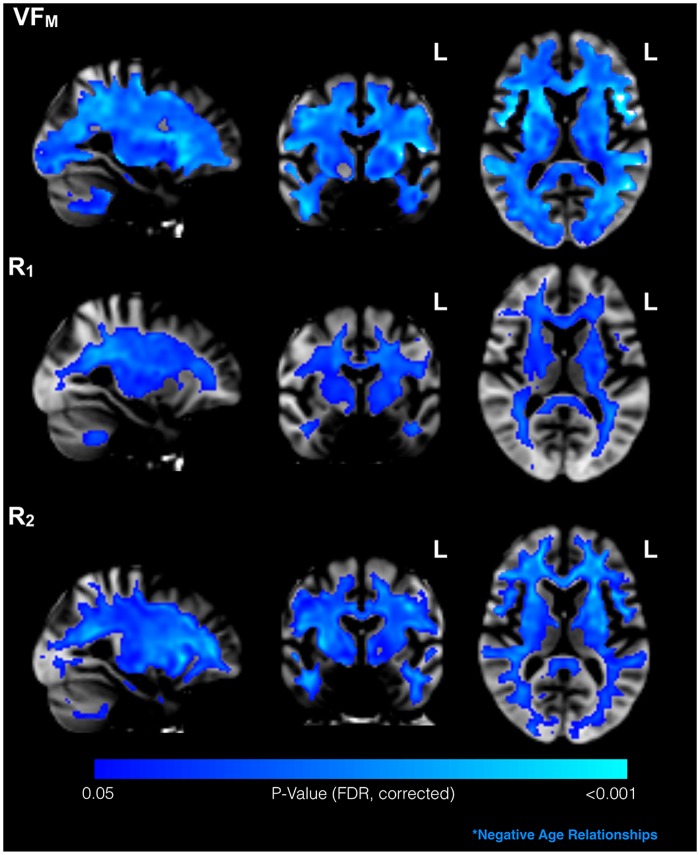
Significant associations between relaxometry measures and age. The negative effect of age on VF_M_, R_1_, and R_2_ was widespread throughout cerebral white matter in this middle- to late- age sample (p<0.05, cluster-corrected).

Extensive age-related changes were observed within this middle-aged sample (Fig 3). In particular, R_1_, R_2_, and VF_M_ were all found to be negatively related with age across the much of the cerebral white matter.

#### Age-by-Group Interaction

**Fig 4:**
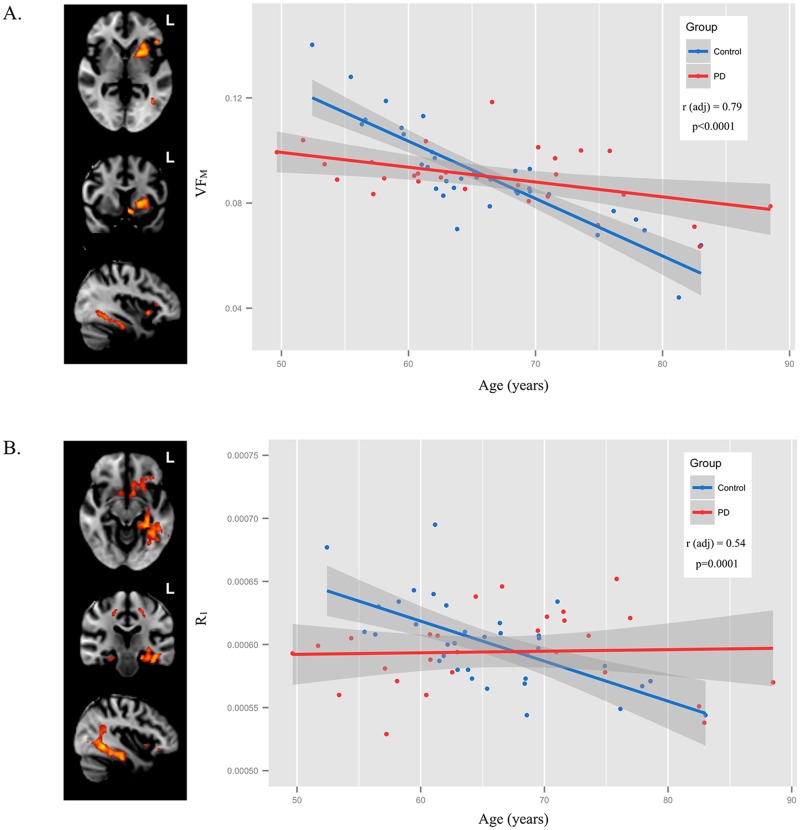
Group differences in the relationship of age to VF_M_. A. Differences in the relationship of age to VF_M_ (i.e. age-by-group interaction) between PD and control subjects were primarily localized to the left putamen left inferior longitudinal fasciculus. B. Significant age-by-group interactions were additionally found with R_1_ within left fusiform gyrus, subcallosal cortex, and orbitofrontal white matter.

Significant VF_M_ age-by-group interactions involved the left insular cortex, extreme capsule, and anterior portion of the putamen and the left temporal, occipital, and fusiform white matter as well as the left parahippocampal gyrus (p<0.05, cluster-corrected). R_1_ age-by-group interactions were observed in the left frontal orbital and subcallosal cortices, among other regions. To illustrate the observed age-by-group interaction, the mean VF_M_ and R_1_ from all significant clusters was calculated for each individual of the PD and control group and plotted against age (Fig 4). The PD group had a higher VF_M_ and R_1_ at advanced ages in comparison to the control group, suggesting a slowing of age-related VF_M_ decline in these regions. Additional brain regions in which a positive age-by-group interaction was found are described in Table 3.

**Table 3:** Brain regions in which a significant (p<0.05, cluster-corrected) positive age-by-group interaction was observed. Shown are the anatomic location, MNI coordinates, t-statistic, and cluster extent. Abbreviations: L: Left; WM: White Matter; Post: Posterior; Ant: Anterior; Inf: Inferior, Mid: Middle.

Image Modality	Location	MNI Coordinates			T-Statistic	Size (mm^3^)
		X	Y	Z		
VF_M_	L. Putamen	-26	22	0	3.60	4336
	L. Inf. Longitudinal fasciculus	-34	-44	-14	3.20	1544
	R. Temporal Occipital Fusiform WM^a^	28	-46	18	3.45	480
	R. Precentral Gyrus WM^a^	20	-20	64	3.81	480
	R. Mid. Temporal Gyrus WM^a^	62	-20	-14	3.16	464
R_1_	L. Uncinate Fasciculus	-30	12	-8	3.13	2280
	L. Inf. Longitudinal fasciculus	-42	-20	-16	3.40	1296
	L. Corticospinal Tract	-18	-18	50	3.23	1216
	L. Sagittal Stratum	-38	-50	-6	3.85	1184
	L. Stria Terminalis	-28	-30	-2	3.30	1136
	L. Frontal Medial WM	-8	46	-18	3.73	752
	R. Cerebellum^a^	12	-56	-40	3.05	440
Post-hoc	L. Putamen	-24	20	2	4.50	6232
	L. Inf. Longitudinal fasciculus	-32	-46	-10	3.88	2248
	R. Tapetum	30	-48	14	3.98	2128
	L. Post. Thalamic Radiation	-28	-62	16	3.23	968
	R. Precentral Gyrus WM	20	-20	64	4.00	616
	R. Mid. Temporal Gyrus WM^a^	62	-18	-16	3.37	584
	L. Thalamus^a^	-8	-20	0	3.03	496
	L. Precentral Gyrus WM^a^	-8	-14	60	2.94	448

^a^Contiguous clusters of at least 50 voxels found to be marginally significant (p<0.1, cluster-corrected)

#### Post-hoc Analysis of VF_M_, R_1_, and R_2_ Measurements

**Fig 5:**
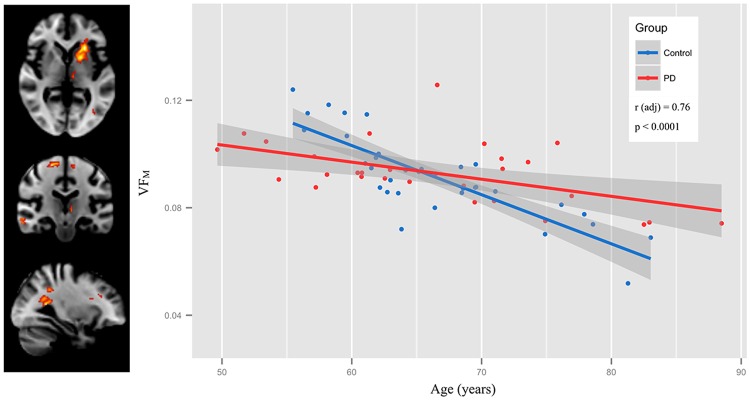
Age-by-group differences correcting for R_1_ and R_2_. Including R_1_ and R_2_ in the age-by-group interaction model for VF_M_ did not alter the significant interaction of the left putamen. To illustrate these age-by-group interactions, mean VF_M_ and R_1_ were calculated from the significant clusters for each individual and plotted as a function of age amd group (PD and age-matched control).

Using a multimodal integrative image analysis approach [45], linear regressions examining the group differences and age-by-group interactions were re-examined by including voxel-wise estimates of R_1_ and R_2_ as additional regressors. Results from this analysis indicated that significant group differences and age-by-group effects on VF_M_ remained; however, the spatial distribution of these differences were moderately altered (Fig 2D, Table 2). For the differences between PD and age-matched controls, significant clusters that initially included the thalamus (Fig 2A) were no longer significant after including R_1_ and R_2_ into the linear regressions, suggesting that the initially observed VF_M_ differences may be primariliy due to alterations in R_1_ and R_2_. However, increased VF_M_ in the right frontal white matter and corpus callosum within the PD group remained, indicating these differences result from underlying changes in myelin content. The age-by-group VF_M_ effects involving the left anterior putamen, anterior limb of internal capusure, and the right middle temporal gyrus remained significant after accounting for R_1_ and R_2_ (Fig 5).

### Associations Between Brain Imaging Parameters and Disease Severity Measures

**Fig 6:**
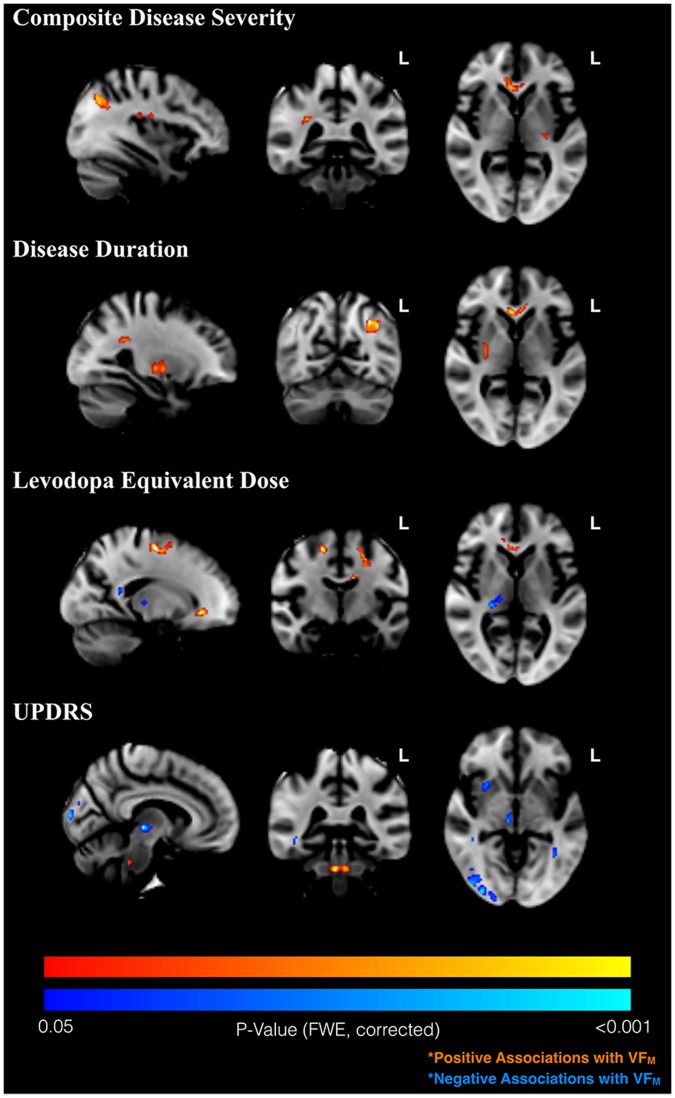
Associations between VF_M_ and Parkinson’s disease severity measures. Significant (p<0.05, cluster-corrected) correlations between VF_M_ and disease severity measures, including disease severity composite (first row), disease duration (second row), daily levodopa equivalent dose levels (third row) and UPDRS score (fourth row). Positive associations (increased VF_M_ with increased clinical score) are shown in red, while negative associations (decreased VF_M_ with increased clinical score) are shown in blue.

As expected, shared variance was present within the disease severity measures; in particular, daily levodopa equivalent dose was found to be partially correlated with UPDRS (pr = 0.45; p<0.05) and disease duration (pr = 0.69; p<0.001), controlling for age, sex, and education as well as the other disease severity measures. As shown in Fig 6, we observed associations between VF_M_ and measures of disease severity within the PD subject group. The composite disease severity measure and disease duration were found to be positively associated with VF_M_ in the genu of the corpus callosum, left hemispheric, and lateral occipital lobule white matter, among other regions. UPDRS scores were found to be positively associated with the VF_M_ of the dorsal midbrain, but negatively correlated with VF_M_ elsewhere, suggesting that within the PD group, higher UPDRS scores may predict lower myelin content. Levodopa equilvalent dose levels were found to be positively related to VF_M_ in several neocortical white matter regions, including left lateral occipital white matter and the genu of the corpus callosum, with the exception of negative correlations in the right thalamus and splenium of the corpus callosum. Greater disease duration was also associated with higher VF_M_ in the right putamen and occipital white matter, and marginally associated with the right posterior corona radiata. A summary of findings is provided in Table 4.

**Table 4:** Brain regions in which significant (p<0.05, cluster-corrected) associations between VF_M_ and disease severity measures were observed. Abbreviations: R: Right; L: Left; WM: White Matter; Sup: Superior; Inf: Inferior; Ant: Anterior; Pos: Posterior; Mid: Middle; Med: Medial; Lat: Lateral.

Clinical Severity Measure	Location	Direction	MNI Coordinates			T-Statistic	Size (mm^3^)
			X	Y	Z		
Composite	L. Lat. Occipital WM	Positive	-30	-64	40	4.26	1416
	Genu of Corpus Callsoum and R. Ant. Corona Radiata	Positive	20	32	-6	3.88	840
	R. Sup. Longitudinal Fasciculus	Positive	30	-36	26	3.47	600
	L. Sup. Longitudinal Fasciculus^a^	Positive	-32	-14	22	3.00	448
	L. Retrolenticular Internal Capsule^a^	Positive	-28	-24	12	3.76	448
Disease Duration	L. Lat. Occipital WM	Positive	-30	-64	40	4.57	1568
	R. Putamen	Positive	28	-14	-2	3.26	808
	Genu of Corpus Callosum^a^	Positive	8	28	0	4.13	584
	R. Post. Corona Radiata^a^	Positive	30	-38	26	3.26	504
LDOPA	L. Lat. Occipital WM	Positive	-30	-64	40	3.65	1304
	Genu of Corpus Callsoum and R. Ant. Corona Radiata	Positive	20	32	-6	4.38	960
	R. Precentral Gyrus WM	Positive	18	-12	58	4.64	752
	R. Superior Longitudinal Fasciculus	Positive	30	-36	26	3.68	736
	Body of Corpus Callosum	Positive	-12	-12	28	4.00	592
	L. Sup. Frontal Gyrus WM^a^	Positive	-22	-8	48	3.99	560
	L. Sup. Longitudinal Fasciculus^a^	Positive	-44	-46	28	3.71	424
	R. Thalamus^a^	Negative	24	-28	6	-3.88	456
	Splenium of Corpus Callosum^a^	Negative	22	-46	16	-4.02	440
UPDRS	Dorsal Midbrain	Positive	4	-34	-36	4.70	616
	R. Lat. Occipital WM	Negative	32	-84	10	-4.37	5976
	L. Lat. Occipital WM	Negative	-26	-84	8	-4.18	2312
	R. Sagittal Stratum^a^	Negative	40	-30	-14	-3.96	576
	L. Post. Thalamic Radiation^a^	Negative	-34	-54	0	-3.98	568
	R. Thalamus^a^	Negative	10	-22	-2	-4.27	472
	R. External Capsule^a^	Negative	28	14	-2	-3.21	424

^a^Contiguous clusters of at least 50 voxels found to be marginally significant (p<0.1, cluster-corrected)

## Discussion

The present study investigated differences in R_1_, R_2_, and VF_M_, a surrogate for myelin content, in individuals with PD compared to age-matched controls, and to what degree VF_M_ was associated with PD severity measures, such as disease duration, levodopa equilvalent dose, and UPDRS total scores. Our findings suggest that PD and/or its treatment effects neuroimaging measures of brain myelin content and that these alterations are related to the severity of clinical symptoms. These findings, although preliminary, are the first to reveal *in vivo* alterations of VF_M_ associated with Parkinson’s disease, and will reinforce and complement a growing body of literature that has described both macro- and microstructural white matter abnormalities in PD [8–12].

Findings from previous studies have suggested that PD has complex effects on white matter microstructure. Many studies have reported microstructural alterations that are considered typical of neurodegeneration, including reduced fractional anisotropy (FA) and increased mean, axial, and radial diffusivities [10,46–48]. While these alterations are thought to reflect losses of white matter microstructure due to deymyelination, axonal damage, and indirect alterations of white matter due to gray matter loss [48], recent evidence has emerged to suggest the white matter microstructure to undergo compensatory or neuroplastic changes during the progression of PD. For example, DTI measures of FA were found to be increased in white matter motor pathways [48], and tracts surrounding the substantia nigra [49]. Our current findings of higher VF_M_, R_1_, and R_2_ in PD may be consistent with this latter framework, however, it is possible that the differential effects of PD on microstructural indices depends on functional connections and neuroanatomic location.

The neural mechanisms underlying these possible compensatory alterations remains unclear. Studies have postulated regional increases in FA may be due increased density of axonal packing [48], but the heightened VF_M_ in precentral gyrus WM observed in this study suggests that compensatory changes may be related to increases in myelin. Such changes of myelin content would be consistent with increases in FA [49], though further research examining such biological changes. Moreover, the age-by-group interaction for the left putamen (Figs 4 and 5) and positive correlations between disease duration and VF_M_ (Fig 6) suggest neuroplasticity alterations may ameliorate age-related decline in VF_M_. Differences observed between previous studies and the current one could be related to dissimilarities in patient characteristics—however, studies of PD tend to involve subjects with a Hoehn and Yahr stage between 1 and 3 and who are additionally able to tolerate study procedures while safely taken off anti-Parkinson medications for 12 hours. The current sample consists of patients with similar Hoehn and Yahr stages as these previous studies [10, 45–49], as well as patients with similar levels of Ldopa to the studies reporting increases in white matter microstructure indices [48,49].

In the present study, correlations between daily Ldopa dose and higher VF_M_ in frontal subcortical regions suggest that anti-Parkinson medications may affect VF_M_. Several of the regions that showed group differences lie within frontal-subcortical and fronto-cerebellar circuits whose function is thought to be critical to movement timing, scaling, and coordination, and communicate at the level of the thalamus [50]—therefore, as neurotransmission and brain activity is known to influence myelination [51–53], altered activity within these circuits may influence VF_M_ over time. In PD, loss of nigrostriatal dopaminergic projections emanating from the SNc results in dopamine deficiency in the striatum (caudate nucleus and putamen), whereas pharmacologic supplementation with dopamine precursors (Ldopa) and agonists can “overdose” limbic and frontal regions, potentially producing psychiatric and cognitive side effects. Moreover, a recent functional connectivity study of PD patients showed that striato-cortical connectivity increases were mediated by levodopa [54]. Thus, VF_M_ increases in PD may also represent in part an adaptive mechanism or side effect to levodopa supplementation in regions of relative dopamine excess.

Although little is known about the effect of dopamine replacement therapies on myelin content in humans, animal studies suggest that dopamine signaling contributes to myelination. During striatal maturation, D1 and D2 receptor expression is followed by a “striking myelination event” and sharp up-regulation of myelin-related genes [55]. Myelin basic protein expression is increased in the striatum of rats with nigrostriatal pathway lesions who receive levodopa supplementation [56], while differentiated rat cortical oligodendrocytes express D2 and D3 dopamine mRNA and are protected from oxidative toxicity by dopamine agonists, suggesting that dopamine receptor activation affects myelination. Atypical antipsychotics, which antagonize dopamine (D_2_) and serotonin (5-HT_2_) receptors [57] also appear to increase or preserve myelination [15,58]. The regional distribution of dopaminergic radiotracers in the brain is correlated with dopamine receptor levels [59]. Therefore, it is possible that increased VF_M_ in the thalamus, frontal, and temporal cortices and connections (such as the genu of corups callosum) we observed in this study reflect the effects of anti-Parkinson medications on brain myelin content. In the PD sample, higher levodopa doses were associated with higher VF_M_ in the genu of corpus callosum, which connects with the relatively dopamine-rich cingulate gyrus^56^.

With regard to the relationship between VF_M_ and disease severity measures, higher VF_M_ in the PD group was distributed primarily in WM tracks that comprise frontal subcortical (superior longitudinal fasciculus) or frontal interhemispheric (genu of corpus callosum) as well as occipital lobe (splenium, posterior thalamic radiation) connections. These relationships commensurate with the observed age-by-group interaction, in which PD patients show preserved VF_M_ with aging. Many of these frontal WM tracks (such as SLF) comprise frontal subcortical connections that are hypothesized to be over- or under- active in PD, and contribute to both motor and cognitive executive symptoms. Visual abnormalities in PD are thought to be caused by loss of retinal pigments [60], but could have trickle down effects in visual and related white matter pathways. Negative associations observed between UPDRS and bilateral occipital and right thalamic white matter may be representative of such secondary effects. Further, such white matter alterations within occipital and thalamic white matter support the hypothesis that visual processing deficits observed in Parkinson’s disease are partially central in origin [61]. The negative relationship between daily Ldopa dose and VF_M_ thalamus may be related to iron accumulation, however, additional research is needed. The partial collinearity among disease severity measures may also be related to sign changes in the relationships between VF_M_ and the individual measures; thus, the relationships between the severity composite and VF_M_ may be the most reliable.

Analysis of multicomponent relaxation aims to distinguish the microstructural contributions of multiple water environments [43,62,63], thereby providing a more sensitive and biologically-specific measure of myelin content. Indeed, while single component R_1_ and R_2_ relaxation rates [28] are highly sensitive to the underlying microstucture, these parameters, along with measures from DTI and magnetization transfer imaging (MTI), may reflect additional pathology, such as edema/inflammation [64,65], alterations of tissue architecture [13,62] and iron content [43,66]. This lack of specificity makes it challenging to draw conclusions about the underlying biology and mechanisms from these parameters alone. Quantification of the VF_M_ has been shown to qualitatively agree with histological assessments of myelin content [67], while other myelin water fraction mapping techniques, such as multi-echo spin-echo approaches [68], have been shown to strongly correlate with histological myelin measurements [69,70]. While mcDESPOT-derived VF_M_ maps may not be considered equivalent to those derived from multi-echo spin-echo approaches [71], these studies suggest that the myelin water fraction may provide improved myelin specificity. Moreover, combining the analyses of VF_M_, R_1_, and R_2_ into a single framework aims to disentangle possible confounding influences. Additional analyses combining VF_M_, R_1_, and R_2_ with other imaging measures, such as those acquired from DTI or MTI, may add insight about the relationship of myelin content to other microstructural changes that accompany the pathogensis of PD and therefore are likely to be beneficial for future investigations.

Although the current study suggests that mcDESPOT has promise as a candidate biomarker for myelin in PD, it has several limitations, the first being its cross-sectional design. Future longitudinal studies are needed to understand intra-individual variation as well as the trajectory of myelin alterations during disease progression. Another limitation is the uncertainty of the effects of levodopa and other medications given to Parkinson’s patients on myelin and water content. Few medication-naïve patients were enrolled in this study, therefore, confident statements about the possible confounding effects of anti-PD medications cannot be made and will require altered study designs. Finally, while mcDESPOT has been shown to provide strong qualitative agreement between histology [67], future studies are needed for histological validation of mcDESPOT. Nonetheless, the literature of studies that have used mcDESPOT [34,37,38,72–77], give assurance that mcDESPOT-derived VF_M_ maps are, at least, strongly sensitive to myelin content.

## Conclusions

The current study provides intruiguing results in line with previous work that has hypothesized oligodendrocytes involvement in the pathogenesis of PD; raises new questions about the role of myelin to subsequent PD pathology, and the extent to which targeted PD medications and therapies alter the topographic changes with disease progression.
